# Supplementary material for: Polypharmacy occurrence and the related risk of premature death among older adults in Denmark: A nationwide register-based cohort study
Source: PLoS One. 2022 Feb 23;17(2):e0264332. doi: 10.1371/journal.pone.0264332 (PMC8865634; doi:10.1371/journal.pone.0264332)
Supplement: S1 Table — (DOCX) [file pone.0264332.s001.docx]

| **Table S1.** Algorithms for identification of chronic conditions | | | |
| --- | --- | --- | --- |
| **Chronic condition** | **ICD10** | **Definition** | **Time period in years** |
| Diabetes | E10, E11 | (DIAG)^a^ and/or (MEDICINE)^b^ all prescriptions with either ATC: A10A, A10B except A10AE56, A10BJ02. | 10 |
| Osteoporosis | M80- M82 | (DIAG)^a^ and/or (MEDICINE)^b^ all prescriptions with either ATC: M05BA01, M05BA04, M05BA07, M05BB01, M05BB03, M05BX03, G03XC01, H05AA02, H05AA03. | From 1995 |
| Diseases of the thyroid | E00-E04, E06, E07 | (DIAG)^a^ and/or (MEDICINE)^b^ all prescriptions with ATC: H03. | 5 |
| Heart disease and heart failure | I20-I25, I50, I11, I13 | (DIAG)^a^ and/or (MEDICINE)^b^ all prescriptions with either ATC: C01A, C01B, C01D, C01E, C09A. | 10 |
| Pulmonary heart disease and diseases of pulmonary | I26, I27, I28 | (DIAG)^a^ | 5 |
| Atrial fibrillation | I48 | (DIAG)^a^ | 10 |
| Aortic and mitral valve disease | I05, I06, I34, I35 | (DIAG)^a^ | 5 |
| Atherosclerosis | I70 | (DIAG)^a^ | 10 |
| Phlebitis | I80 | (DIAG)^a^ | 5 |
| Hypertension | I10, I12, I15 | (DIAG)^a^ and/or (MEDICINE)^b^ all prescriptions with either ATC: C02A, C02B, C02C, C02DA, C02L, C03A, C03B, C03D, C03E, C03X, C07C, C07D, C08G, C09BA, C09DA, C09XA52 C02DB, C02DD, C02DG, C07, C07F, C08, C09BB, C09DB, C09. | 5 |
| High cholesterol | E780, E782, E784, E785 | (DIAG)^a^ and/or (MEDICINE)^b^ all prescriptions with ATC: C10. | 5 |
| Inflammatory intestinal diseases | K50, K51 | (DIAG)^a^ | From 1995 |
| Colon irritable (IBS) | K58 | (DIAG)^a^ | 10 |
| Diseases of liver, biliary tract and pancreas | K71-K77, K861, K87 | (DIAG)^a^ | 10 |
| Stroke | G45-G46, I60-I69 | (DIAG)^a^ | 10 |
| Epilepsy | G40, G41 | (DIAG)^a^ and/or (MEDICINE)^b^ all prescriptions with either ATC: N03, N05BA, N05CD.  Note: For prescriptions with N05BA and N05CD indication codes 155 or 753 should be registered. | 5 |
| Migraine and other headache syndromes | G43, G44 | (DIAG)^a^ and/or (MEDICINE)^b^ all prescriptions with ATC: N02C. | 10 |
| Dementia | F00-F03, G30, G318B, G318E, G319, G310B | (DIAG)^a^ and/or (MEDICINE)^b^ all prescriptions with ATC: N06D and/or (MEDICINE)^b^ all prescriptions with identification codes 329, 330 or 331. | From 1995 |
| Parkinson | G20- G22, F023 | (DIAG)^a^ and/or (MEDICINE)^b^ all prescriptions with ATC: N04. | From 1995 |
| Sclerosis | G35 | (DIAG)^a^ | From 1995 |
| Chronic Obstructive Pulmonary Disease | J40-J44, J47, J96 | (DIAG)^a^ and/or (MEDICINE)^b^ all prescriptions with either ATC: R03AC18, R03AC19, R03AL02, R03AL03, R03AL04, R03AL05, R03AL06, R03AL09, R03BB04, R03BB05, R03BB06, R03BB07, R03DX07 and/or (MEDICINE)^b^ all prescriptions with identification codes 379 or 464. | From 1995 |
| Asthma | J45 | (DIAG)^a^ and/or (MEDICINE)^b^ all prescriptions with ATC: R03DC03 and/or (MEDICINE)^b^ all prescriptions with identification codes 379 or 464. | From 1995 |
| Chronic kidney disease | N18 | (DIAG)^a^ | From 1995 |
| Cancer digestive organs | C15-C26 | (DIAG)^a^ | 10 |
| Cancer in bronchus, lungs and thoracic area | C30-C39 | (DIAG)^a^ | 10 |
| Melanoma cancer of skin | C43 | (DIAG)^a^ | 10 |
| Breast cancer | C50 | (DIAG)^a^ | 10 |
| Cancer in the reproductive organs | C51-C58, C60-C63 | (DIAG)^a^ | 10 |
| Other cancers (not metastasis) | C00-C14, C40,C41, C45-C49, C64-C75, C81-C96 | (DIAG)^a^ | 10 |
| Depression | F32, F33, F341, F0632 | (DIAG)^a^ and/or (MEDICINE)^b^ all prescriptions with ATC: N06A.  Note: All prescription should be registered with indication code 168. | 5 |
| Anxiety | F401, F411 | (DIAG)^a^ and/or (MEDICINE)^b^ all prescriptions with identification codes 163 or 371. | 10 |
| Schizophrenia, schizotypal mental illness, paranoid psychoses | F20-F22, F25, F28, F29 | (DIAG)^a^ and/or (MEDICINE)^b^ all prescriptions with ATC: N05AX13, N05AX12, N05AH03, N05AX08. | From 1995 |
| Bipolar affective disorder | F30, F31 | (DIAG)^a^ and/or (MEDICINE)^b^ all prescriptions with ATC: N05A, N06A.  Note: All prescription should be registered with indication code 491 or 631. | From 1995 |
| Post-traumatic stress disorder | F431 | (DIAG)^a^ | From 1995 |
| Obsessive compulsive disorder | F42 | (DIAG)^a^ and/or (MEDICINE)^b^ all prescriptions with ATC: N06A.  Note: All prescription should be registered with indication code 472 or 596. | 10 |
| Eating disorder | F50 | (DIAG)^a^ | 5 |
| After-effects/ states of alcohol dependence | G312, G621, G721, K292, K70, K860 | (DIAG)^a^ | From 1995 |
| Allergies | J301-J304 | (DIAG)^a^ and/or (MEDICINE)^b^ all prescriptions with ATC: V01AA02, V01AA03, V01AA05, V01AA11, R01AC, R01AD, R06A, S01G, R01BA52. | From 1995 |
| HIV/AIDS | B20- B24 | (DIAG)^a^ | From 1995 |
| Joint disease | M05, M060, M068, M070, M071, M073, M100, M109 | (DIAG)^a^ and/or (MEDICINE)^b^ all prescriptions with identification codes 147, 402 or 641. | From 1995 |
| Osteoarthritis | M15-M19 | (DIAG)^a^ | From 1995 |
| Back pain | M40-M43, M45-M54 | (DIAG)^a^ | From 1995 |
| Fibromyalgia | M797 | (DIAG)^a^ | From 1995 |
| Damage to the spinal cord, spinal trauma and syndromes with paralysis | G81-G83, S14, S24, S34, T093 | (DIAG)^a^ | From 1995 |
| Blindness | H54 | (DIAG)^a^ | From 1995 |
| Tinnitus | H931 | (DIAG)^a^ | From 1995 |
| Congenital malformations | Q0-Q9 | (DIAG)^a^ | From 1995 |
| ^a^ DIAG: All patients who have had at least one hospital or outpatient contact with one of the ICD-10 codes specified for the condition within the time period specified for the condition prior to study entrance. Both ongoing and finalized contacts as well as primary (A), secondary (B) and additional (+) diagnosis are included.  ^b^ MEDICINE: All patients that have had at least two prescriptions for medicine with the ATC codes and/or identification codes specified for the condition with a period of two years prior to study entrance. | | | |
