# Supplementary material for: Polypharmacy occurrence and the related risk of premature death among older adults in Denmark: A nationwide register-based cohort study
Source: PLoS One. 2022 Feb 23;17(2):e0264332. doi: 10.1371/journal.pone.0264332 (PMC8865634; doi:10.1371/journal.pone.0264332)
Supplement: S2 Table — (DOCX) [file pone.0264332.s002.docx]

| **Table S2.** Population characteristics across polypharmacy status before and after applying weights (N = 1,338,058). | | | | | | | | | | | |
| --- | --- | --- | --- | --- | --- | --- | --- | --- | --- | --- | --- |
|  | **Unweighted** | | | | |  | **Weighted** | | | | |
|  | **No  polypharmacy** | |  | **Polypharmacy** | |  | **No  polypharmacy** | |  | **Polypharmacy** | |
|  | **N** | **%** |  | **N** | **%** |  | **N^*^** | **%** |  | **N^*^** | **%** |
| All | 1,011,941 | 100 |  | 833,235 | 100 |  | 382,420 | 100 |  | 382,420 | 100 |
| **Sex** |  |  |  |  |  |  |  |  |  |  |  |
| Male | 479,571 | 47.4 |  | 375,752 | 35.6 |  | 177,244 | 46.3 |  | 177,244 | 46.3 |
| Female | 532,370 | 52.6 |  | 457,483 | 21.3 |  | 205,196 | 53.7 |  | 205,196 | 53.7 |
| **Age** |  |  |  |  |  |  |  |  |  |  |  |
| 65–69 years | 610,631 | 60.3 |  | 296,584 | 35.6 |  | 176,852 | 46.2 |  | 176,852 | 46.2 |
| 70–74 years | 172,870 | 17.1 |  | 177,782 | 21.3 |  | 78,888 | 20.6 |  | 78,888 | 20.6 |
| 75–79 years | 108,020 | 10.7 |  | 143,030 | 17.2 |  | 55,815 | 14.6 |  | 55,815 | 14.6 |
| 80–84 years | 64,829 | 6.4 |  | 105,528 | 12.7 |  | 36,774 | 9.6 |  | 36,774 | 9.6 |
| 85–89 years | 36,450 | 3.6 |  | 69,837 | 8.4 |  | 22,086 | 5.8 |  | 22,086 | 5.8 |
| 90–94 years | 15,092 | 1.5 |  | 31,590 | 3.8 |  | 9,411 | 2.5 |  | 9,411 | 2.5 |
| 95+ years | 4,049 | 0.4 |  | 8,884 | 1.1 |  | 2,612 | 0.7 |  | 2,612 | 0.7 |
| **Region of residence** |  |  |  |  |  |  |  |  |  |  |  |
| Northern Jutland Region | 109,022 | 10.8 |  | 95,531 | 11.5 |  | 42,304 | 11.1 |  | 42,304 | 11.1 |
| Mid Jutland Region | 218,165 | 21.6 |  | 183,411 | 22.0 |  | 82,959 | 21.7 |  | 82,959 | 21.7 |
| Region of Southern Denmark | 227,178 | 22.4 |  | 191,083 | 22.9 |  | 86,825 | 22.7 |  | 86,825 | 22.7 |
| Capital Region of Denmark | 288,101 | 28.5 |  | 228,009 | 27.4 |  | 107,081 | 28.0 |  | 107,081 | 28.0 |
| Region Zealand | 169,475 | 16.7 |  | 135,201 | 16.2 |  | 63,271 | 16.5 |  | 63,271 | 16.5 |
| **Migration status** |  |  |  |  |  |  |  |  |  |  |  |
| Danish | 958,623 | 94.7 |  | 801,986 | 96.2 |  | 365,872 | 95.7 |  | 365,872 | 95.7 |
| Western migrant | 30,124 | 3.0 |  | 17,471 | 2.1 |  | 9,067 | 2.4 |  | 9,067 | 2.4 |
| Non-Western migrant | 23,194 | 2.3 |  | 13,778 | 1.7 |  | 7,501 | 2.0 |  | 7,501 | 2.0 |
| **Marital Status** |  |  |  |  |  |  |  |  |  |  |  |
| Married | 632,917 | 62.5 |  | 459,203 | 55.1 |  | 225,040 | 58.8 |  | 225,040 | 58.8 |
| Divorced | 137,150 | 54.9 |  | 112,598 | 45.1 |  | 52,480 | 13.7 |  | 52,480 | 13.7 |
| Widowed | 171,116 | 16.9 |  | 214,848 | 25.7 |  | 81,374 | 21.3 |  | 81,374 | 21.3 |
| Never married | 70,758 | 7.0 |  | 46,950 | 5.6 |  | 23,545 | 6.2 |  | 23,545 | 6.2 |
| **Highest achieved education** |  |  |  |  |  |  |  |  |  |  |  |
| No education | 30,801 | 0.3 |  | 29,774 | 3.6 |  | 12,238 | 3.2 |  | 12,238 | 3.2 |
| Secondary school | 340,019 | 33.6 |  | 355,058 | 42.6 |  | 147,659 | 38.6 |  | 147,659 | 38.6 |
| High school/skilled education | 403,967 | 39.9 |  | 304,292 | 36.5 |  | 146,923 | 38.4 |  | 146,923 | 38.4 |
| Short higher education | 31,463 | 3.1 |  | 20,120 | 2.4 |  | 10,338 | 2.7 |  | 10,338 | 2.7 |
| Middle higher education | 149,135 | 14.7 |  | 92,509 | 11.1 |  | 48,253 | 12.6 |  | 48,253 | 12.6 |
| High higher education | 56,5566 | 5.6 |  | 31,482 | 3.8 |  | 17,028 | 4.5 |  | 17,028 | 4.5 |
| **Income** |  |  |  |  |  |  |  |  |  |  |  |
| First quartile | 211,257 | 20.9 |  | 243,227 | 29.2 |  | 96,040 | 25.1 |  | 96,040 | 25.1 |
| Second quartile | 231,272 | 22.9 |  | 233,390 | 28.0 |  | 99,517 | 26.0 |  | 99,517 | 26.0 |
| Third quartile | 269,819 | 26.7 |  | 196,226 | 23.5 |  | 97,365 | 25.5 |  | 97,365 | 25.5 |
| Fourth quartile | 295,929 | 29.2 |  | 158,592 | 19.0 |  | 88,441 | 23.1 |  | 88,441 | 23.1 |
| Unknown | 3,664 | 0.2 |  | 1,800 | 0.2 |  | 1,077 | 0.3 |  | 1,077 | 0.3 |
| **Number of chronic conditions** |  |  |  |  |  |  |  |  |  |  |  |
| 0-1 | 509,600 | 50.4 |  | 161,182 | 19.3 |  | 114,133 | 29.8 |  | 114,133 | 29.8 |
| 2+ | 502,341 | 49.6 |  | 672,053 | 80.7 |  | 268,307 | 70.2 |  | 268,307 | 70.2 |
| **Year of inclusion** |  |  |  |  |  |  |  |  |  |  |  |
| 2013 | 744,445 | 73.6 |  | 729,924 | 87.6 |  | 314,958 | 82.4 |  | 314,958 | 82.4 |
| 2014 | 67,582 | 6.7 |  | 31,862 | 3.8 |  | 20,155 | 5.3 |  | 20,155 | 5.3 |
| 2015 | 67,776 | 6.7 |  | 29,145 | 3.5 |  | 17,961 | 4.7 |  | 17,961 | 4.7 |
| 2016 | 65,596 | 6.5 |  | 23,992 | 2.9 |  | 15,357 | 4.0 |  | 15,357 | 4.0 |
| 2017 | 66,542 | 6.6 |  | 18,312 | 2.2 |  | 14,007 | 3.7 |  | 14,007 | 3.7 |
| ^*^ The frequencies in the weighted population are calculated based on weights.  Note: Polypharmacy was in the mortality analysis defined as a time-dependent variable. Therefore participants who were not exposed to polypharmacy at first by were exposed later, contributes to both the exposed and unexposed population. Hence, N in the non-weighted population exceeds 1,338,058. | | | | | | | | | | | |
