# Supplementary material for: Polypharmacy occurrence and the related risk of premature death among older adults in Denmark: A nationwide register-based cohort study
Source: PLoS One. 2022 Feb 23;17(2):e0264332. doi: 10.1371/journal.pone.0264332 (PMC8865634; doi:10.1371/journal.pone.0264332)
Supplement: S3 Table — (DOCX) [file pone.0264332.s003.docx]

| **Table S3.** Population characteristics across excessive polypharmacy status before and after applying weights (N = 1,338,058). | | | | | | | | | | | |
| --- | --- | --- | --- | --- | --- | --- | --- | --- | --- | --- | --- |
|  | **Unweighted** | | | | |  | **Weighted** | | | | |
|  | **No excessive polypharmacy** | |  | **Excessive polypharmacy** | |  | **No excessive polypharmacy** | |  | **Excessive polypharmacy** | |
|  | **N** | **%** |  | **N** | **%** |  | **N^*^** | **%** |  | **N^*^** | **%** |
| All | 1,284,350 | 100 |  | 287,366 | 100 |  | 205,451 | 100 |  | 205,451 | 100 |
| **Sex** |  |  |  |  |  |  |  |  |  |  |  |
| Male | 597,796 | 46.5 |  | 123,999 | 43.2 |  | 90,219 | 44.0 |  | 90,219 | 44.0 |
| Female | 686,554 | 53.5 |  | 163,367 | 56.8 |  | 115,132 | 56.0 |  | 115,132 | 56.0 |
| **Age** |  |  |  |  |  |  |  |  |  |  |  |
| 65–69 years | 676,917 | 52.7 |  | 68,712 | 23.9 |  | 58,420 | 28.4 |  | 58,420 | 28.4 |
| 70–74 years | 232,521 | 18.1 |  | 57,249 | 19.9 |  | 42,555 | 20.7 |  | 42,555 | 20.7 |
| 75–79 years | 162,608 | 12.7 |  | 55,444 | 19.3 |  | 38,526 | 18.8 |  | 38,526 | 18.8 |
| 80–84 years | 108,590 | 8.5 |  | 48,524 | 16.9 |  | 31,478 | 15.3 |  | 31,478 | 15.3 |
| 85–89 years | 66,892 | 5.2 |  | 35,516 | 12.4 |  | 21,877 | 10.6 |  | 21,877 | 10.6 |
| 90–94 years | 28,963 | 2.3 |  | 17,332 | 6.0 |  | 9,904 | 4.8 |  | 9,904 | 4.8 |
| 95+ years | 7,859 | 0.6 |  | 4,589 | 1.6 |  | 2,696 | 1.3 |  | 2,696 | 1.3 |
| **Region of residence** |  |  |  |  |  |  |  |  |  |  |  |
| Northern Jutland Region | 141,862 | 11.0 |  | 34,362 | 12.0 |  | 24,107 | 11.7 |  | 24,107 | 11.7 |
| Mid Jutland Region | 278,721 | 21.7 |  | 67,066 | 23.3 |  | 46,975 | 22.9 |  | 46,975 | 22.9 |
| Region of Southern Denmark | 291,442 | 22.7 |  | 65,801 | 22.9 |  | 47,095 | 22.9 |  | 47,095 | 22.9 |
| Capital Region of Denmark | 360,288 | 28.1 |  | 75,613 | 26.3 |  | 54,913 | 26.7 |  | 54,913 | 26.7 |
| Region Zealand | 212,037 | 16.5 |  | 44,524 | 15.5 |  | 32,360 | 15.7 |  | 32,360 | 15.7 |
| **Migration status** |  |  |  |  |  |  |  |  |  |  |  |
| Danish | 1,222,063 | 95.2 |  | 276,710 | 96.3 |  | 197,436 | 96.1 |  | 197,436 | 96.1 |
| Western migrant | 35,477 | 2.8 |  | 5,697 | 2.0 |  | 4,269 | 2.1 |  | 4,269 | 2.1 |
| Non-Western migrant | 26,810 | 2.1 |  | 4,959 | 1.7 |  | 3,746 | 1.8 |  | 3,746 | 1.8 |
| **Marital Status** |  |  |  |  |  |  |  |  |  |  |  |
| Married | 767,337 | 59.7 |  | 138,975 | 48.4 |  | 103,825 | 50.5 |  | 103,825 | 50.5 |
| Divorced | 171,169 | 20.4 |  | 90,343 | 31.4 |  | 29,772 | 14.5 |  | 29,772 | 14.5 |
| Widowed | 261,435 | 13.3 |  | 42,306 | 14.7 |  | 60,238 | 29.3 |  | 60,238 | 29.3 |
| Never married | 84,409 | 6.6 |  | 15,742 | 5.5 |  | 11,616 | 5.7 |  | 11,616 | 5.7 |
| **Highest achieved education** |  |  |  |  |  |  |  |  |  |  |  |
| No education | 43,951 | 3.4 |  | 611,510 | 4.0 |  | 8,064 | 3.9 |  | 8,064 | 3.9 |
| Secondary school | 471,175 | 36.7 |  | 141,696 | 49.3 |  | 96,689 | 47.1 |  | 96,689 | 47.1 |
| High school/skilled education | 494,582 | 38.5 |  | 95,533 | 33.2 |  | 70,759 | 34.4 |  | 70,759 | 34.4 |
| Short higher education | 36,964 | 2.9 |  | 5,624 | 2.0 |  | 4,309 | 2.1 |  | 4,309 | 2.1 |
| Middle higher education | 173,374 | 13.5 |  | 25,008 | 8.7 |  | 19,335 | 9.4 |  | 19,335 | 9.4 |
| High higher education | 64,304 | 5.0 |  | 7,995 | 2.8 |  | 6,395 | 3.1 |  | 6,395 | 3.1 |
| **Income** |  |  |  |  |  |  |  |  |  |  |  |
| First quartile | 310,683 | 24.2 |  | 102,399 | 35.6 |  | 68,804 | 33.5 |  | 68,804 | 33.5 |
| Second quartile | 315,352 | 24.6 |  | 89,108 | 31.0 |  | 62,092 | 30.2 |  | 62,092 | 30.2 |
| Third quartile | 324,880 | 25.3 |  | 58,963 | 20.5 |  | 44,671 | 21.8 |  | 44,671 | 21.8 |
| Fourth quartile | 329,589 | 25.7 |  | 36,397 | 12.7 |  | 29,483 | 14.4 |  | 29,483 | 14.4 |
| Unknown | 3,846 | 0.2 |  | 499 | 0.2 |  | 402 | 0.2 |  | 402 | 0.2 |
| **Number of chronic conditions** |  |  |  |  |  |  |  |  |  |  |  |
| 0-1 | 521,971 | 40.6 |  | 21,512 | 7.5 |  | 20,051 | 9.8 |  | 20,051 | 9.8 |
| 2+ | 762,379 | 59.4 |  | 265,854 | 92.5 |  | 185,400 | 90.2 |  | 185,400 | 90.2 |
| **Year of inclusion** |  |  |  |  |  |  |  |  |  |  |  |
| 2013 | 1,016,854 | 79.2 |  | 264,740 | 92.1 |  | 187,046 | 91.0 |  | 187,046 | 91.0 |
| 2014 | 76,582 | 5.3 |  | 7,825 | 2.7 |  | 5,516 | 2.7 |  | 5,516 | 2.7 |
| 2015 | 67,776 | 5.3 |  | 6,563 | 2.3 |  | 4,890 | 2.4 |  | 4,890 | 2.4 |
| 2016 | 65,596 | 5.1 |  | 5,027 | 1.7 |  | 4,158 | 2.0 |  | 4,158 | 2.0 |
| 2017 | 66,542 | 5.2 |  | 3,211 | 1.1 |  | 3,841 | 1.9 |  | 3,841 | 1.9 |
| ^*^ The frequencies in the weighted population are calculated based on weights. | | | | | | | | | | | |
