# Supplementary material for: Polypharmacy occurrence and the related risk of premature death among older adults in Denmark: A nationwide register-based cohort study
Source: PLoS One. 2022 Feb 23;17(2):e0264332. doi: 10.1371/journal.pone.0264332 (PMC8865634; doi:10.1371/journal.pone.0264332)
Supplement: S4 Table — (DOCX) [file pone.0264332.s004.docx]

| **Table S4.** Percentage of medications taken by those exposed at baseline. | | | | | | |
| --- | --- | --- | --- | --- | --- | --- |
| **1^st^ and 2^nd^ ATC level** | **Kind of medication** | **Polypharmacy** (N = 326,117) |  | | **Excessive polypharmacy (**N = 53,708) | |
|  |  | **%** | |  | | **%** |
| **A** | **Alimentary tract and metabolism** | **61.0** | |  | | **91.2** |
| A01 | Stomatological preparations | 0,2 | |  | | 0,4 |
| A02 | Drugs for acid related disorders | 33,9 | |  | | 66,2 |
| A03 | Drugs for functional gastrointestinal disorders | 1,2 | |  | | 4,0 |
| A04 | Antiemetics and anti-nauseants | 0,0 | |  | | 0,1 |
| A06 | Drugs for constipation | 7,3 | |  | | 21,6 |
| A07 | Antidiarrheals, intestinal anti-inflammatory/anti-infective agents | 1,3 | |  | | 2,6 |
| A08 | Anti-obesity preparations, excl. Diet products | 0,1 | |  | | 0,2 |
| A09 | Digestives, incl. Enzymes | 0,2 | |  | | 0,3 |
| A10 | Drugs used in diabetes | 28,4 | |  | | 55,2 |
| A11 | Vitamins | 0,5 | |  | | 0,9 |
| A12 | Mineral supplements | 18,4 | |  | | 44,1 |
| **B** | **Blood and blood-forming organs** | **68.9** | |  | | **83.5** |
| B01 | Antithrombotic agents | 74,8 | |  | | 99,3 |
| B02 | Antihemorrhagics | 0,0 | |  | | 0,1 |
| B03 | Anti-anaemic preparations | 12,7 | |  | | 27,3 |
| **C** | **Cardiovascular system** | **95.0** | |  | | **98.5** |
| C01 | Cardiac therapy | 15,0 | |  | | 37,5 |
| C02 | Antihypertensives | 2,7 | |  | | 4,6 |
| C03 | Diuretics | 60,5 | |  | | 97,8 |
| C04 | Peripheral vasodilators | 0,0 | |  | | 0,0 |
| C05 | Vasoprotectives | 0,3 | |  | | 0,6 |
| C07 | Beta-blocking agents | 29,7 | |  | | 42,6 |
| C08 | Calcium channel blockers | 42,3 | |  | | 49,0 |
| C09 | Agents acting on the renin-angiotensin system | 62,8 | |  | | 67,5 |
| C10 | Lipid-modifying agents | 60,1 | |  | | 72,4 |
| **D** | **Dermatologicals** | **0.9** | |  | | **1.6** |
| D01 | Antifungals for dermatological use | 0,5 | |  | | 1,0 |
| D02 | Emollients and protectives | 0,0 | |  | | 0,0 |
| D04 | Anti-pruritics, incl. antihistamines, anaesthetics, etc. | 0,0 | |  | | 0,0 |
| D05 | Anti-psoriatics | 0,1 | |  | | 0,1 |
| D06 | Antibiotics and chemotherapeutics for dermatological use | 0,0 | |  | | 0,0 |
| D07 | Corticosteroids, dermatological preparations | 0,3 | |  | | 0,7 |
| D10 | Anti-acne preparations | 0,0 | |  | | 0,0 |
| D11 | Other dermatological preparations | 0,0 | |  | | 0,0 |
| **G** | **Genito urinary system and sex hormones** | **15.3** | |  | | **22.9** |
| G01 | Gynaecological anti-infectives and antiseptics | 0,0 | |  | | 0,1 |
| G02 | Other gynaecologicals | 0,0 | |  | | 0.0 |
| G03 | Sex hormones and modulators of the genital system | 4,6 | |  | | 6,7 |
| G04 | Urologicals | 13,0 | |  | | 20,7 |
| **H** | **Systemic hormonal preparations ex. Sex hormones** | **13.4** | |  | | **26.3** |
| H01 | Pituitary and hypothalamic hormones and analogues | 0,1 | |  | | 0,3 |
| H02 | Corticosteroids for systemic use | 5,9 | |  | | 14,6 |
| H03 | Thyroid therapy | 7,9 | |  | | 13,7 |
| H05 | Calcium homeostasis | 0,2 | |  | | 0,3 |
| **J** | **Anti-infectives for systemic use** | **6.0** | |  | | **14.2** |
| J01 | Anti-bacterials for systemic use | 6,3 | |  | | 15,0 |
| J02 | Antimycotics for systemic use | 0,1 | |  | | 0,4 |
| J04 | Anti-mycobacterials | 0,0 | |  | | 0,1 |
| J05 | Antivirals for systemic use | 0.0 | |  | | 0,1 |
| **L** | **Antineoplastic and immunomodulating agents** | **1.6** | |  | | **2.7** |
| L01 | Antineoplastic agents | 0.0 | |  | | 0.0 |
| L02 | Endocrine therapy | 0,1 | |  | | 0,3 |
| L04 | Immunosuppressants | 1,5 | |  | | 2,4 |
| **M** | **Musculoskeletal system** | **22.7** | |  | | **38.0** |
| M01 | Anti-inflammatory and antirheumatic products | 9,8 | |  | | 15,6 |
| M02 | Topical products for joint and muscular pain | 0,0 | |  | | 0,1 |
| M03 | Muscle relaxants | 0,7 | |  | | 2,2 |
| M04 | Antigout preparations | 3,5 | |  | | 7,6 |
| M05 | Drugs for treatment of bone diseases | 10,7 | |  | | 18,2 |
| M09 | Other drugs for disorders of the musculoskeletal system | 0,0 | |  | | 0,0 |
| **N** | **Nervous system** | **53.7** | |  | | **84.1** |
| N01 | Anaesthetics | 0,0 | |  | | 0,0 |
| N02 | Analgesics | 37,2 | |  | | 83,8 |
| N03 | Antiepileptics | 4,2 | |  | | 11,4 |
| N04 | Anti-Parkinson drugs | 1,9 | |  | | 3,4 |
| N05 | Psycholeptics | 18,6 | |  | | 41,3 |
| N06 | Psychoanaleptics | 31,4 | |  | | 63,7 |
| N07 | Other nervous system drugs | 1,0 | |  | | 2,5 |
| **P** | **Anti-parasitic products, insecticides and repellents** | **0.5** | |  | | **1.5** |
| P01 | Anti-protozoals | 0,5 | |  | | 1,5 |
| P02 | Anthelmintics | 0,0 | |  | | 0,0 |
| P03 | Ectoparasiticides | 0,0 | |  | | 0.0 |
| **R** | **Respiratory system** | **23.8** | |  | | **42.8** |
| R01 | Nasal preparations | 2,4 | |  | | 3,9 |
| R02 | Throat preparations | 0,0 | |  | | 0,1 |
| R03 | Drugs for obstructive airway diseases | 29,1 | |  | | 63,5 |
| R05 | Cough and cold preparations | 2,8 | |  | | 6,6 |
| R06 | Antihistamines for systemic use | 4,6 | |  | | 9,9 |
| **S** | **Sensory system** | **6.6** | |  | | **9.0** |
| S01 | Ophthalmologicals | 8.8 | |  | | 12,2 |
| **V** | **Various** | **0.0** | |  | | **0.0** |
| V01 | Allergens | 0,0 | |  | | 0.0 |
| V03 | All other therapeutic products | 0,0 | |  | | 0,0 |
| V07 | All other non-therapeutic products | 0,0 | |  | | 0,0 |
