# Supplementary material for: Polypharmacy occurrence and the related risk of premature death among older adults in Denmark: A nationwide register-based cohort study
Source: PLoS One. 2022 Feb 23;17(2):e0264332. doi: 10.1371/journal.pone.0264332 (PMC8865634; doi:10.1371/journal.pone.0264332)
Supplement: S5 Table — (DOCX) [file pone.0264332.s005.docx]

| **Table S5.** Overview of ATC codes excluded in the sensitivity analysis | |
| --- | --- |
| **Type of medicine** | **ATC code** |
| All antibiotics | J: Anti-infectives for systemic use  A01AB: Anti-infectives and antiseptics for local oral treatment  A02BD: Combinations for eradication of Heliobactor pylori  A07A: Intestinal anti-infectives  B05CA: Anti-infectives  D01: antifungals for dermatological use  D06A: Antibiotics for topical use  D07C: Corticosteroids, combinations with antibiotics  D09AA: Ointment dressings with anti-infectives  D10AF: Anti-infectives for treatment of acne  G01: Gynaecological anti-infectives and antiseptics  All codes starting with P: Antiparasitic products, insecticides and repellents  R02AB: Antibiotics  S01A: Anti-infectives  S01C_ Anti-inflammatory agents and anti-infectives in combination  S02A: Anti-infectives  S02C: Corticosteroids and anti-infectives in combination  S03A: Anti-infectives  S03C: Corticosteroids and anti-infectives in combination |
| Medicine and products used for diagnostic purposes or other non-therapeutic purposes | V04: Diagnostic agents (e.g. urine tests, tests for diabetes etc.)  V07: All other non-therapeutic products (e.g. plasters, stoma equipment, cosmetics etc.)  V08: Contrast media used for X-rays, MRI and ultrasound  V09: Diagnostic radiopharmaceuticals  V20: Surgical dressings |
| Dermatological products with no specific therapeutic effect or use | D09: Medicated dressings  D02: Emollients and protectives |
