# Supplementary material for: Polypharmacy occurrence and the related risk of premature death among older adults in Denmark: A nationwide register-based cohort study
Source: PLoS One. 2022 Feb 23;17(2):e0264332. doi: 10.1371/journal.pone.0264332 (PMC8865634; doi:10.1371/journal.pone.0264332)
Supplement: S6 Table — (DOCX) [file pone.0264332.s006.docx]

| **Table S6.** Mean medication use and proportion of polypharmacy cases in the main analysis and sensitivity analysis. | | | | | | | | |
| --- | --- | --- | --- | --- | --- | --- | --- | --- |
|  | **Sensitivity analysis** | | |  | **Main analysis** | | |  |
|  | Mean medication use | Polypharmacy cases (%) | Excessive polypharmacy cases (%) |  | Mean medication use | Polypharmacy cases (%) | Excessive polypharmacy cases (%) |  |
| All | 3.2 | 28.6 | 4.4 |  | 3.3 | 29.0 | 4.7 |  |
| **Sex** |  |  |  |  |  |  |  |  |
| Male | 3.1 | 27.5 | 4.0 |  | 3.1 | 27.7 | 4.0 |  |
| Female | 3.3 | 29.6 | 4.7 |  | 3.4 | 30.2 | 5.2 |  |
| **Age** |  |  |  |  |  |  |  |  |
| 65–69 years | 2.6 | 20.7 | 3.1 |  | 2.5 | 20.2 | 2.8 |  |
| 70–74 years | 3.2 | 27.9 | 3.9 |  | 3.3 | 29.0 | 4.5 |  |
| 75–79 years | 3.9 | 36.6 | 5.7 |  | 4.0 | 37.9 | 6.4 |  |
| 80–84 years | 4.4 | 43.8 | 7.2 |  | 4.5 | 45.3 | 8.3 |  |
| 85–89 years | 4.7 | 49.0 | 8.2 |  | 4.9 | 50.8 | 9.6 |  |
| 90–94 years | 4.9 | 50.5 | 8.1 |  | 5.1 | 53.0 | 9.8 |  |
| 95+ years | 4.7 | 49.9 | 8.1 |  | 5.0 | 52.7 | 8.3 |  |
| **Region of residence** |  |  |  |  |  |  |  |  |
| Northern Jutland Region | 3.5 | 31.7 | 5.3 |  | 3.5 | 31.9 | 5.5 |  |
| Mid Jutland Region | 3.3 | 29.9 | 5.0 |  | 3.4 | 30.2 | 5.2 |  |
| Region of Southern Denmark | 3.3 | 29.5 | 4.5 |  | 3.3 | 29.9 | 4.8 |  |
| Capital Region of Denmark | 3.0 | 26.4 | 3.7 |  | 3.1 | 27.0 | 4.0 |  |
| Region Zealand | 3.1 | 27.3 | 4.0 |  | 3.2 | 27.7 | 4.2 |  |
| **Migration status** |  |  |  |  |  |  |  |  |
| Danish | 3.2 | 28.9 | 4.4 |  | 3.3 | 29.4 | 4.7 |  |
| Western migrant | 2.4 | 20.2 | 3.1 |  | 2.4 | 20.7 | 3.3 |  |
| Non-Western migrant | 2.6 | 23.6 | 4.1 |  | 2.6 | 23.6 | 3.9 |  |
| **Marital Status** |  |  |  |  |  |  |  |  |
| Married | 2.9 | 24.5 | 3.3 |  | 2.9 | 24.7 | 3.4 |  |
| Divorced | 3.3 | 30.0 | 5.6 |  | 3.4 | 30.3 | 5.8 |  |
| Widowed | 4.1 | 39.8 | 6.7 |  | 4.2 | 41.1 | 7.6 |  |
| Never married | 2.9 | 26.4 | 4.3 |  | 2.9 | 26.3 | 4.3 |  |
| **Highest achieved education** |  |  |  |  |  |  |  |  |
| No education | 3.4 | 34.0 | 5.1 |  | 3.6 | 35.5 | 6.0 |  |
| Secondary school | 3.8 | 35.7 | 6.2 |  | 3.9 | 36.4 | 6.7 |  |
| High school/skilled education | 3.0 | 26.1 | 3.6 |  | 3.0 | 26.2 | 3.7 |  |
| Short higher education | 2.6 | 21.3 | 2.6 |  | 2.6 | 21.5 | 2.6 |  |
| Middle higher education | 2.5 | 20.1 | 2.5 |  | 2.6 | 20.3 | 2.6 |  |
| High higher education | 2.2 | 17.0 | 2.0 |  | 2.3 | 17.2 | 2.0 |  |
| **Income** |  |  |  |  |  |  |  |  |
| First quartile | 3.9 | 37.6 | 6.4 |  | 4.0 | 38.5 | 7.1 |  |
| Second quartile | 3.7 | 34.6 | 5.9 |  | 3.8 | 35.3 | 6.3 |  |
| Third quartile | 3.0 | 25.1 | 3.5 |  | 3.0 | 25.3 | 3.5 |  |
| Fourth quartile | 2.3 | 17.0 | 1.8 |  | 2.3 | 17.0 | 1.8 |  |
| Unknown | 1.8 | 15.1 | 3.0 |  | 1.8 | 15.2 | 3.1 |  |
| **Number of chronic conditions** |  |  |  |  |  |  |  |  |
| 0-1 | 1.0 | 2.6 | 0.0 |  | 1.1 | 2.8 | 0.1 |  |
| 2+ | 4.6 | 45.2 | 7.2 |  | 4.7 | 45.8 | 7.6 |  |
| **Year of inclusion** |  |  |  |  |  |  |  |  |
| 2013 | 3.4 | 30.6 | 4.6 |  | 3.5 | 31.7 | 5.2 |  |
| 2014 | 2.5 | 18.1 | 3.6 |  | 2.6 | 18.1 | 3.6 |  |
| 2015 | 2.5 | 18.4 | 3.5 |  | 2.6 | 18.5 | 3.5 |  |
| 2016 | 2.5 | 18.5 | 3.6 |  | 2.6 | 18.6 | 3.6 |  |
| 2017 | 2.5 | 17.9 | 3.5 |  | 2.6 | 18.3 | 3.5 |  |
