# Supplementary material for: Polypharmacy occurrence and the related risk of premature death among older adults in Denmark: A nationwide register-based cohort study
Source: PLoS One. 2022 Feb 23;17(2):e0264332. doi: 10.1371/journal.pone.0264332 (PMC8865634; doi:10.1371/journal.pone.0264332)
Supplement: Attachment — Submitted filename: Response to Reviewers.docx [file pone.0264332.s008.docx]

**Response to Reviewer**

We appreciate the feedback and comments that we have received from the reviewer. In this document we respond to the comment given and describe the changes that we have made to the manuscript. We hope that these modifications will satisfy the reviewer.

| **Comment** | **Response** |
| --- | --- |
| Thank you to the authors for addressing the points I raised. Although I still have some reservations over the manner in which the results are presented, I feel the clarifications that have been made help address these concerns.  My only outstanding (minor) remark is the response to my comment 2 (methods). The response was generally fine, but the statement "limitations include that large weights are given for individual participants, large variability in the estimated treatment effect may be observed and bias" seems to be incomplete, although it may be simply a grammatical issue. I think it perhaps should read "limitations include that large weights are given for individual participants, large variability in the estimated treatment effect may be observed, and THERE MAY BE bias"? (if so, bias in what sense?) Or alternatively was the term "and bias" supposed to be deleted and the word "and" added prior to "although"? | Thank you so much for your positive feedback on the revised manuscript. Your suggestions have definitely helped us improve it.  The one comment you give to our revised manuscript surely warrants some attention. We acknowledge that the word bias cannot be added without further explanation. We therefore propose another sentence to replace the one in question (p. 8, lines 155-158). |
